# Ethanolic Extracts of Adlay Testa and Hull and Their Active Biomolecules Exert Relaxing Effect on Uterine Muscle Contraction through Blocking Extracellular Calcium Influx in Ex Vivo and In Vivo Studies

**DOI:** 10.3390/biom11060887

**Published:** 2021-06-15

**Authors:** Yun-Ju Huang, Yu-Chieh Chen, Hsin-Yuan Chen, Yi-Fen Chiang, Mohamed Ali, Wenchang Chiang, Cheng-Pei Chung, Shih-Min Hsia

**Affiliations:** 1 School of Nutrition and Health Sciences, College of Nutrition, Taipei Medical University, Taipei 11031, Taiwan; d04641004@ntu.edu.tw (Y.-J.H.); hsin246@gmail.com (H.-Y.C.); yvonne840828@gmail.com (Y.-F.C.); 2 Institute of Food Science and Technology, National Taiwan University, Taipei 10617, Taiwan; r00641033@ntu.edu.tw (Y.-C.C.); chiang@ntu.edu.tw (W.C.); 3 Clinical Pharmacy Department, Faculty of Pharmacy, Ain Shams University, Cairo 11566, Egypt; mohamed.aboouf@pharma.asu.edu.eg; 4 Department of Nutrition and Health Sciences, Chang Gung University of Science and Technology, Taoyuan 33303, Taiwan; d95641002@ntu.edu.tw; 5 Graduate Institute of Metabolism and Obesity Sciences, College of Nutrition, Taipei Medical University, Taipei 11031, Taiwan; 6 School of Food Safety, College of Nutrition, Taipei Medical University, Taipei 11031, Taiwan; 7 Nutrition Research Center, Taipei Medical University Hospital, Taipei 11031, Taiwan

**Keywords:** dysmenorrhea, *Coix lachryma-jobi*, smooth muscle, oxytocin-induced writhing, acetic acid writhing

## Abstract

Dysmenorrhea is one of the most prevalent disorders in gynecology. Historically, adlay (*Coix lachryma-jobi* L. var. Ma-yuen Stapf.) has been explored for its anti-tumor, pain relief, anti-inflammatory, and analgesic effects. The aim of this study was to evaluate the effects of adlay seeds on the inhibition of uterine contraction and thus dysmenorrhea relief, in vitro and in vivo. HPLC-MS and GC were used to elucidate the ethyl acetate fraction of adlay testa ethanolic extract (ATE-EA) and ethyl acetate fraction of adlay hull ethanolic extract (AHE-EA). Elucidation yielded flavonoids, phytosterols, and fatty acids. Uterine leiomyomas and normal adjacent myometrial tissue were evaluated by oxytocin- and PG-induced uterine contractility. ATE-EA and AHE-EA suppressed uterine contraction induced by prostaglandin F2 alpha (PGF_2α_), oxytocin, carbachol, and high-KCl solution ex vivo. In addition, the external calcium (Ca^2+^) influx induced contraction, and increased Ca^2+^ concentration was inhibited by ATE-EA and AHE-EA on the uterine smooth muscle of rats. Furthermore, ATE-EA and AHE-EA effectively attenuated the contraction of normal human myometrium tissues more than adjacent uterine leiomyoma in response to PGF_2α_. 3,5,6,7,8,3′,4′-Heptamethoxyflavone and chrysoeriol produced a remarkable inhibition with values of IC_50_ = 24.91 and 25.59 µM, respectively. The experimental results showed that treatment with ATE-EA at 30 mg/day effectively decreased the writhing frequency both on the oxytocin-induced writhing test and acetic acid writhing test of the ICR mouse.

## 1. Introduction

Dysmenorrhea is the most common gynecologic disorder in women of reproductive age, with a prevalence rate ranges from 16% to 91% [1]. Dysmenorrhea has two types: primary and secondary. Primary dysmenorrhea is defined as painful menstruation, without pelvic disease, and significant symptoms such as sweating, vomiting, fatigue, back pain, headaches, and diarrhea; notably, severe pain does not commonly occur. Secondary dysmenorrhea refers to a pathological pelvic condition with painful menstruation [2]. Meta-regression analysis reported that dysmenorrhea had higher rates of pain than dyspareunia and noncyclical pain in quality studies [3]. Dysmenorrhea is among the leading causes with negative impact on absenteeism, limitations in daily living and socialization, and healthcare [4]. Risk factors of dysmenorrhea include weight [5], duration of menstrual flow [5,6,7], earlier age at menarche [5,6,7], cigarette smoking [5,6,7,8], and family history of dysmenorrhea [7,9]. Interestingly, severity of dysmenorrhea was not related to the length of menstrual cycle, weight, and age. Moreover, some psychological factors are associated with increased risk of dysmenorrhea such as depression, anxiety, stress, and somatic complaints [9]. Treatment of dysmenorrhea include using non-steroidal anti-inflammatory drugs (NSAIDs) (38.5%) and oral pills (37.0%) to relieve menstrual pain [4]. Some previous studies have reported that NSAIDs were not clearly proven with superior efficacy, and the side effect of gastrointestinal upset is common [10,11]. Lifestyle adjustments, diet, nutrient supplements, herbs, and complementary and alternative medicine are being explored as treatment options for dysmenorrhea.

The adlay (*Coix Lacryma-Jobi*, Job’s tears) seed is a traditional Chinese medicine that has been widely used in Asian countries for thousands of years. They have been recognized for their diverse physiological activity such as anti-inflammatory, anti-obesity, anti-hyperlipidemia, anti-tumor, and anti-allergy effects in recent studies [12]. These health benefits of adlay extracts are in part attributed to their unique phytochemical components including total phenolics and total flavonoids. For example, these phytochemicals contributed to antioxidant activity and proliferation inhibition on human liver cancer cells [13]. Moreover, polyphenol extracts of adlay exert a cardioprotective effect via decreased serum levels of TC, LDL-C, and increased HDL-C [14]. Our previous study showed that the ethyl acetate fraction of adlay hull significantly inhibited uterine myometrial hyperplasia in rats [15]. The current study further investigates the inhibitory effect of ethyl acetate fraction of adlay testa on the uterine smooth muscle contraction both in an animal model and in human tissue.

Flavonoids are natural products found in plants, flowers, fruits, and leaves and belong to polyphenolic compounds [16]. Flavonoids possess antioxidant, free radical scavenging, anti-proliferative activities [17], and an anti-apoptotic effect [18] to prevent or treat a variety of diseases such as renal diseases, cancer, cardiovascular disease, neurodegenerative disorders, ulcers, and gastritis [19]. Previous studies have demonstrated that flavonoids from onion effectively attenuated uterine contraction induced by Prostaglandin F2α (PGF_2α_) in rats [20]. In addition, it was reported that the relaxant effect of flavonoid on colon smooth muscle was via blocking calcium (Ca^2+^) influx [21]. Our previous study showed that flavonoids quercetin and naringenin of active compounds have shown inhibitory effects on uterine contractions in rats [22]. Therefore, adlay might show therapeutic potential against dysmenorrhea.

There is little information in the literature regarding the beneficial effect of adlay ethanolic extracts against dysmenorrhea. Most of the previous studies showed that adlay and its fractions focused on its chemopreventive effect [23]. This study aimed to investigate the muscle-relaxing effect of adlay ethanolic extracts on uterine smooth muscle contraction induced by PGF_2α_ using ex vivo and in vivo as well as quantitative estimation of flavonoid, phytosterol, and fatty acid of ethyl acetate fraction of adlay hull and testa.

## 2. Materials and Methods

### 2.1. Plant Material

The Coix seeds were collected by commissioned farmers (No. 4 Coix, Taichung). The plant material was dehulled and separated into adlay hull, adlay testa, and adlay (red Job’s tears) by the air sieve method. Adlay was also further refined and divided into adlay bran and polished adlay for the experiment.

### 2.2. Preparation of Extracts

The plant materials (hull, testa, bran, and polished adlay) were soaked in 10 times the volume (*w*/*v*) of 95% ethanol at room temperature overnight. The filtrate was concentrated under reduced pressure after filtering the impurities and repeatedly extracted from the remaining residue twice. The resultants were collected from four parts referred to as adlay hull ethanolic extract (AHE), adlay testa ethanolic extract (ATE), adlay bran ethanolic extract (ABE), and polished adlay ethanolic extract (PAE).

### 2.3. Preparation of Ethanolic Extract and Its Fractions form Adlay Hull and Adlay Testa

The AHE and ATE with different polarities solvents were prepared by adding 5 times the amount (*w*/*v*) of distilled water and further fractionated through successive extraction with n-hexane, ethyl acetate, and n-butanol. Each fraction of AHE was concentrated to dryness under reduced pressure, which gives 0.22%, 0.1%, 0.07%, and a 0.12% yield in hexane (AHE-Hex), ethyl acetate (AHE-EA), n-butanol (AHE-Bu), and water (AHE-Wa), respectively. The same process was applied on ATE and obtained 1.57%, 0.29%, 0.2%, and 0.67% yields in hexane (ATE-Hex), ethyl acetate (ATE-EA), n-butanol (ATE-Bu), and water (ATE-Wa), respectively. Each fraction of AHE and ATE was prepared as 200 mg/mL stock in dimethyl sulfoxide (DMSO) and stored at 4 °C.

### 2.4. Identification and Quantification of Flavonoids in Ethyl Acetate Fraction of Adlay Hull (AHE-EA) and Ethyl Acetate Fraction of Adlay Testa (ATE-EA) by HPLC-MS (High-Performance Liquid Chromatography–Mass Spectrometry) and HPLC

HPLC coupled to MS was used to identify the flavonoid compounds through their retention times (by comparing them to those of reference standards) and the mass of the selected ions. The analytical HPLC system employed consisted of a Finnign MAT (P4000) high-performance liquid chromatography coupled with a UV–VIS detector (UV2000) and autosampler (AS3000). The separation was achieved on a Gemini C18 3 µm 2 × 150 mm column at ambient temperature. The separation of the extract solution was carried out with a flow of 0.3 mL/min. A 20 µL aliquot of extract solution was injected. The mobile phase consisted of water with 0.01% formic acid (solvent A) and methanol with acetonitrile (solvent B, 1:1 *v*/*v*). The gradient used for the determination of flavonoid was as follows: 80% A/20% B, 0–5 min; 60% A/40% B, 5–10 min; 40% A/60% B, 10–30 min; 35% A/65% B, 30–40 min; 1% A/99% B, 40–50 min. MS was performed on a Finnigan MAT LCQ (Thermoquest Corp, San Jose, CA, USA) equipped with a pneumatically assisted electrospray interface. The spray needle voltage was 5 kV, and the temperature of the heated inlet capillary 250 °C. Nebulizer pressure was 60 psi, and auxiliary gas was 30 psi.

### 2.5. Identification and Quantification of Fatty Acid and Phytosterol in Ethyl Acetate Fraction of Adlay Hull (AHE-EA) and Ethyl Acetate Fraction of Adlay Testa (ATE-EA) by GC

Gas chromatography (GC) analysis was conducted for qualitative analysis of phytosterol. GC was equipped with an FID and a CP-5 megabore capillary column (30 m × 0.53 mm 1 μm; J&W Scientific, Folsom, CA, USA). An initial oven temperature of 272 °C was maintained for 8 min. Then, the temperature was increased to 280 °C at a rate of 1.2 °C/min, and temperature was further increased to 300 °C at a rate of 10 °C/min for 5 min. Then, the final temperature was increased at 20 °C/min to reach 315 °C. 1 μL was injected into the instrument using a split ratio of 80:1. The injector port and detector temperatures were set at 330 °C and 340 °C, respectively. The column flow rate was 5.0 mL/min, and nitrogen was used as the carrier gas. Phytosterol and fatty acid compositions were determined on the basis of the relative chromatographic areas to compare calibration curves of authentic standards to calculate. The fatty acid content was detected by promulgation for confirmation of national standards.

### 2.6. Experimental Animal Studies

All animal studies were conducted according to the protocols approved by the Institutional Animal Care and Use Committee (IACUC) of Taipei Medical University (Permit No. LAC-2016-0167 and LAC-2016-0221). Female Sprague-Dawley (SD) rats were purchased from a commercial supplier (BioLASCO Taiwan Co., Ltd., Taipei, Taiwan). They were kept for 6~8 weeks under constant conditions of temperature (24 ± 2 °C), relative humidity (50~60%), and light and dark cycles of 12 h during the uterine contraction in vitro experiment. Animals were provided with a standard rodent chow diet (Rodent Laboratory Chow #5001, Ralston Purina Co., St. Louis, MO, USA), and the food and water were allowed ad libitum. 

For in vivo uterine contractions experiment, female ICR mice (20–25 g) were purchased from a commercial supplier (BioLASCO Taiwan Co., Ltd., Taipei, Taiwan). The mice were confirmed at the estrous stage by microscopic examination of a vaginal smear in the experiment. The animals were housed and acclimatized under a normal 12 h light-dark cycle, temperature (24 ± 2 °C), and relative humidity (50–70%) for 1 week before the experiments. All mice had ad libitum access to food and water. The mice were then randomly divided into five groups (n = 8 per group) as follows: sesame oil (control), 2 mg/kg bw of stilbestrol (model control), 30 mg/day of AHE-EA, 30 mg/day of ATE-EA, and Ibuprofen 120 mg/kg (positive control) following the third day at the end of the experiment by oral gavage. Mice were treated with stilbestrol, a nonsteroidal estrogen medication, on the first six days in the oxytocin-induced mice writhing model. The mice received 20 IU/kg of oxytocin after treatment at 1 h before the writhing test. 

Regarding the acetic acid-induced abdominal writhing test, mice were randomly separated into the following groups (8 mice/group): 0.5% CMC (control), AHE-EA 30 mg/day, ATE-EA 30 mg/day, and aspirin 200 mg/kg. All mice received only one time treatment by oral gavage 1 h before being induced by an intraperitoneal (i.p.) injection of acetic acid (0.6%).

### 2.7. Oxytocin-Induced Writhing Test

The test was performed on the basis of a previously standardized reference [24]. Female mice were treated with stilbestrol 2 mg/kg by oral gavage for 6 days. The dose of 30 mg/day of AHE-EA and ATE-EA was selected on the basis of an IC_50_ experiment of PGF_2α_ that resulted in a uterine contraction in vitro. AHE-EA, ATE-EA, and ibuprofen 120 mg/kg (positive control) were administered orally, beginning on day 3 and repeated every 24 h until the end of the experiment. Mice were injected intraperitoneally with 20 IU/kg oxytocin solution on day 7. The time that elapsed until the occurrence of the writhing response was recorded as pain latency as well as the number of writhing responses within 30 min after oxytocin injection. After the writhing test, uterine tissue was removed and collected to further analysis.

### 2.8. Acetic Acid-Induced Writhing Test

The acetic acid-induced writhing test was used to assess abdominal contraction on the basis of previous investigations [25]. The modified test included a contraction of the abdominal muscle with an elevating and stretching of the hind limbs [26]. AHE-EA, ATE-EA, and aspirin (200 mg/kg) were administered orally 1 h before providing 10 mL/kg 0.6% acetic acid by intraperitoneal injection. The frequencies and latency of writhing responses were counted and recorded in 30 min.

### 2.9. Tissue Samples

Matched specimens of uterine leiomyoma and adjacent normal myometrium were obtained from women at the Department of Obstetrics and Gynecology at National Taiwan University Hospital from Dr. Lin-Hung Wei laboratory. Dissected specimens were immediately immersed in liquid saline and stored at 4 °C until we performed smooth muscle test contraction within 2 days. This study was approved by the IRB of National Taiwan University Hospital (IRB number: 201210072RIC).

### 2.10. Uterine Preparations and Measurement of Uterine Contraction

Rats and mice were sacrificed by carbon dioxide, and the uteri were removed and placed in Krebs’ solution (113 mM NaCl, 4.8 mM KCl, 2.5 mM CaCl_2_, 18 mM NaHCO_3_, 1.2 mM KH_2_PO_4_, 1.2 mM MgSO_4_, 5.5 mM glucose, 30 mM mannitol). After removing surrounding fats, we cut the uterine tissues into 0.5~1 cm segments of equal length. To maintain the tissue activity, we placed the segments in isolated organ baths containing Krebs’ solution at 37 °C with a 95% O_2_ and 5% CO_2_ supply. Equilibrate with 2 g of weight for at least 30 min for uterine smooth muscle contraction stability, PGF_2α_, oxytocin, and other drugs of smooth muscle contraction were added to each organ bath to measure their effects on uterine smooth muscle. Each concentration of adlay samples was added separately for 10 min in organ baths in a cumulative manner. Amplitude and frequency of uterine contractions were recorded with force displacement transducers by using the LabScribe software.

### 2.11. Statistical Analysis

The results of treatment effects were expressed as mean ± standard deviation (SD). Statistical comparisons were performed using Student’s *t*-test or one-way ANOVA test, followed by Duncan’s multiple range test post hoc analysis using SPSS (IBM Corporation, Armonk, NY, USA), and *p*-values less than or equal to 0.05 were considered significant.

## 3. Results

### 3.1. Effect of Different Adlay Parts and Its Ethanolic Extract Fractions on PGF_2α_-Induced Uterine Hypercontraction Ex Vivo

**Figure 1:**
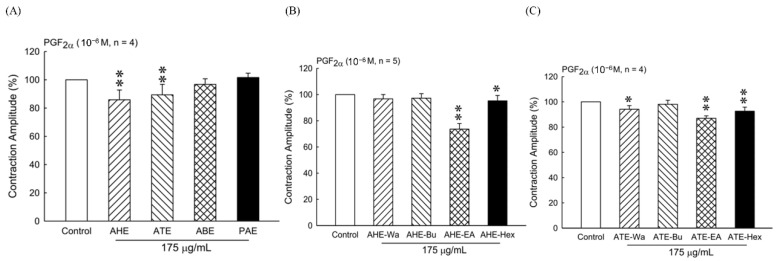
Effect of adlay on the uterine contraction induced by PGF_2α_ (10^−6^ M). (**A**) Effect of different parts of adlay on isolated rat’s uterine contractions. (**B**) Inhibitory effect of AHE subfractions on isolated rat’s uterine contraction. (**C**) Inhibitory effect of ATE subfractions on isolated rat’s uterine contraction. Data represent mean values ± SD. * *p* < 0.05, ** *p* < 0.01 compared with control. AHE, ethanolic extracts of adlay hull; ATE, ethanolic extracts of adlay testa; ABE, ethanolic extracts of adlay bran; PAE, ethanolic extracts of polished adlay; AHE-Wa, H_2_O fraction of adlay hull ethanolic extract; AHE-Bu, 1-butanol fraction of adlay hull ethanolic extract; AHE-EA, ethyl acetate fraction of adlay hull ethanolic extract; AHE-Hex, n-hexane fraction of adlay hull ethanolic extract; ATE-Wa, H_2_O fraction of adlay testa ethanolic extract; ATE-Bu, 1-butanol fraction of adlay testa ethanolic extract; ATE-EA, ethyl acetate fraction of adlay testa ethanolic extract; ATE-Hex, n-hexane fraction of adlay testa ethanolic extract.

These response experiments were performed to determine the effects of adlay ethanolic extracts on the uterine contractions. The four parts of adlay—hull (AHE), testa (ATE), bran (ABE), and polished adlay (PAE)—were tested. Results showed that AHE and ATE inhibited uterine contractions at a dose of 175 µg/mL (Figure 1A). The amplitude of uterine contractions was inhibited by fractions of n-hexane and ethyl acetate of AHE and ATE (Figure 1B,C). Therefore, we used AHE-EA (ethyl acetate fraction of AHE) and ATE-EA (ethyl acetate fraction of ATE) for further investigation in this study.

### 3.2. Effect of ATE-EA and AHE-EA on Uterine Contractions Induced by PGF_2α_, Oxytocin, Carbachol, and High-KCl Solution Ex Vivo

**Figure 2:**
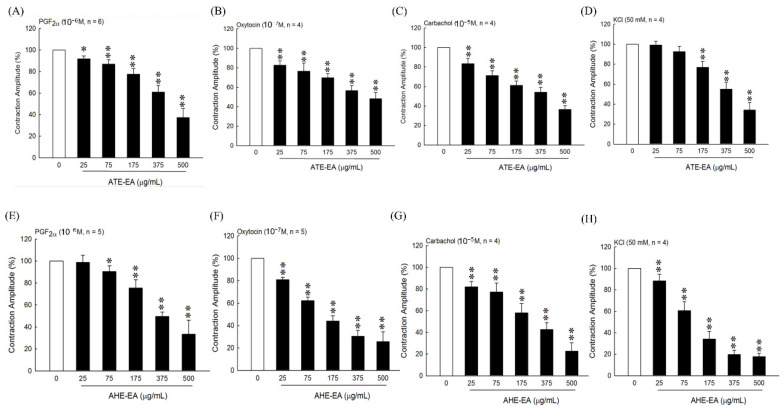
The effect of different concentrations of ATE-EA on uterine contraction amplitude under influence of PGF_2α_ (**A**), oxytocin (**B**), carbachol (**C**), and KCl solution (**D**). The effect of different concentrations of AHE-EA on uterine contraction amplitude under influence of PGF_2α_ (**E**), oxytocin (**F**), carbachol (**G**), and KCl solution (**H**). Data represent mean values ± SD. * *p* < 0.05, ** *p* < 0.01 compared with control. PGF_2α_, prostaglandin F2 alpha; ATE-EA, ethyl acetate fraction of adlay testa ethanolic extract; AHE-EA, ethyl acetate fraction of adlay hull ethanolic extract.

To evaluate whether ATE-EA and AHE-EA inhibited the contraction of uterine contraction by blocking the influx of extracellular calcium, we stimulated uterine strips with agonists including PGF_2α_, oxytocin, and carbachol, which resulted in a marked increase in amplitude and contraction frequency. As shown in Figure 2A–C,E–G, the results showed that ATE-EA and AHE-EA significantly decreased uterine contractile activity in a concentration-dependent manner (25, 75, 175, 375, and 500 µg/mL) under the continued presence of these stimulatory drugs. A high-KCl solution caused depolarization and sustained tonic contraction, which was maintained as long as it was performed. Different concentrations of ATE-EA and AHE-EA progressively reduced uterine contraction in the presence of KCl solution (Figure 2D,H).

### 3.3. Effect of ATE-EA and AHE-EA (175 µg/mL) on Uterine Contractions Induced by Different Concentrations of PGF_2α_, Oxytocin, and Carbachol and High-KCl Solution Ex Vivo

**Figure 3:**
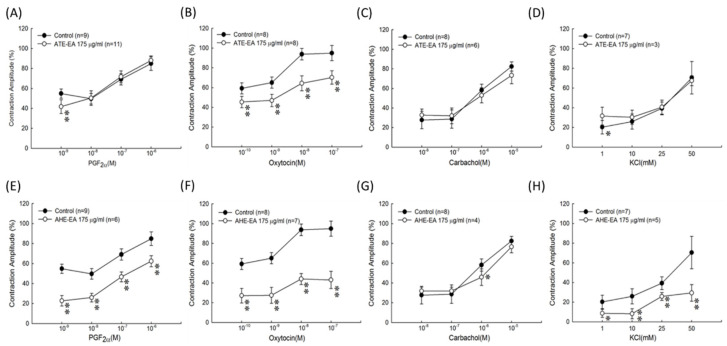
Effects of ATE-EA given cumulatively on the PGF_2α_-induced (**A**), oxytocin-induced (**B**), carbachol-induced (**C**), and KCl solution-induced (**D**) contractions of isolated rat uterus. AHE-EA given cumulatively on the PGF_2α_-induced (**E**), oxytocin-induced (**F**), carbachol-induced (**G**), and KCl solution-induced (**H**) contractions of isolated rat uterus. Data represent mean values ± SD. * *p* < 0.05, ** *p* < 0.01 compared with control. PGF_2α_, prostaglandin F2 alpha; ATE-EA, ethyl acetate fraction of adlay testa ethanolic extract; AHE-EA, ethyl acetate fraction of adlay hull ethanolic extract.

We wanted to confirm the previous findings using a larger concentration scale of used stimulants PGF_2α_, oxytocin, carbachol, and KCl solution. ATE-EA at 175 µg/mL had a preventive effect against oxytocin-induced muscle contraction (Figure 3B). However, ATE-EA failed to inhibit muscle contraction induced by increasing concentration of PGF_2α_, carbachol, and KCl (Figure 3A,C,D). Interestingly, AHE-EA was able to significantly inhibit uterine contraction induced by increasing concentration of all stimulants PGF_2α_, oxytocin, and KCl solution (Figure 3D,E,G) except carbachol (Figure 3F).

### 3.4. Inhibitory Effect of ATE-EA and AHE-EA on Calcium (Ca^2+^)-Dependent Contractile Responses Ex Vivo

**Figure 4:**
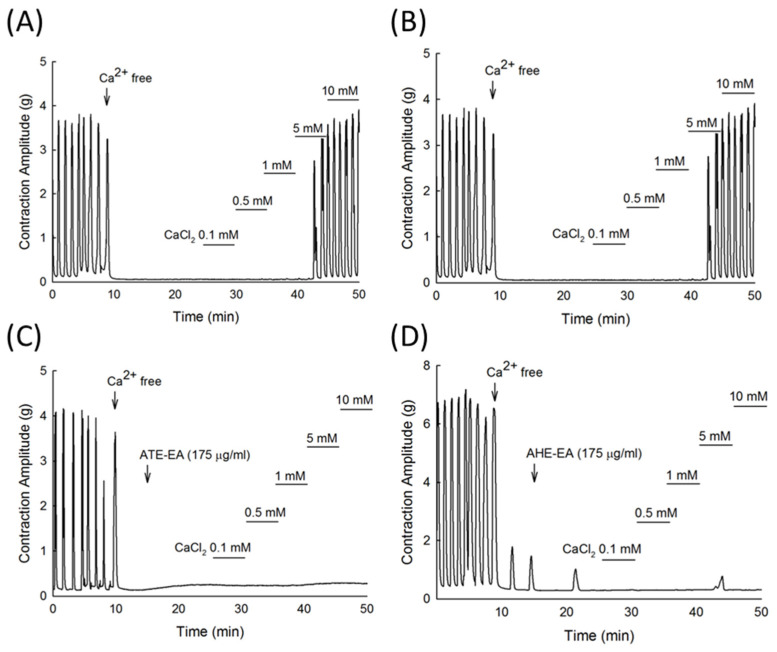
Inhibitory effect of ATE-EA and AHE-EA on calcium (Ca^2+^)-dependent contractile responses ex vivo. (**A**,**B**) Calcium chloride (0.1–10 mM) added into the bathing solution resulted in a concentration-dependent increase in contraction of the isolated rat uterus, which was inhibited by (**C**) ATE-EA (175 μg/mL) and (**D**) AHE-EA (175 μg/mL).

To investigate if the mechanism of action underlying the muscle contraction inhibitory effect of the ATE-EA and AHE-EA involved calcium, we added external calcium as CaCl_2_, which showed a concentration-dependent contractile response (Figure 4A,B) that was inhibited significantly by ATE-EA and AHE-EA, as shown in Figure 4C,D, respectively.

### 3.5. Inhibitory Effect of Flavonoids on PGF2α-Induced Uterine Hypercontraction Ex Vivo

We evaluated the inhibitory effects of the flavonoid on uterine contractions induced by PGF2α. As shown in Table 1, among the tested compounds, 3,5,6,7,8,3′,4′-heptamethoxyflavone and chrysoeriol exhibited remarkable inhibition activities with IC_50_ values of 24.91 and 25.59 μM, respectively. On the other hand, naringenin and liquiritigenin showed marginal inhibitory activities, even at higher concentrations (IC_50_ values 147.1 and 176.8 μM, respectively).

### 3.6. Effect of ATE-EA and AHE-EA on Uterine Contractility in Human Tissue

#### 3.6.1. Dose–Response Effects of ATE-EA and AHE-EA on Uterine Contractions Induced by PGF_2α_, Oxytocin, and Carbachol and High-KCl Solution

**Figure 5:**
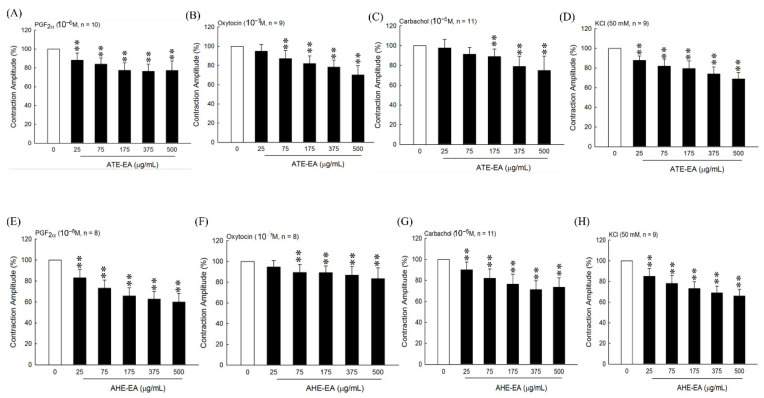
Inhibitory effects of serial concentrations of ATE-EA and AHE-EA on contractions of isolated human uterus induced by PGF_2α_ (**A**,**E**), oxytocin (**B**,**F**), carbachol (**C**,**G**), and KCl solution (**D**,**H**). Data represent mean values ± SD. ** *p* < 0.01 compared with control. PGF_2α_, prostaglandin F2 alpha; ATE-EA, ethyl acetate fraction of adlay testa ethanolic extract; AHE-EA, ethyl acetate fraction of adlay hull ethanolic extract.

Similar to previous findings, human muscle contraction induced by PGF_2α_, oxytocin, and carbachol and high-KCl solution was significantly decreased at high concentrations of ATE-EA (175, 375, and 500 µg/mL) (Figure 5A–D). Similar results were found for using AHE-EA at doses of 75, 175, 375, and 500 µg/mL (Figure 5E–G). 

#### 3.6.2. Effect of ATE-EA and AHE-EA on PGF_2α_-Induced Uterine Contractions

**Figure 6:**
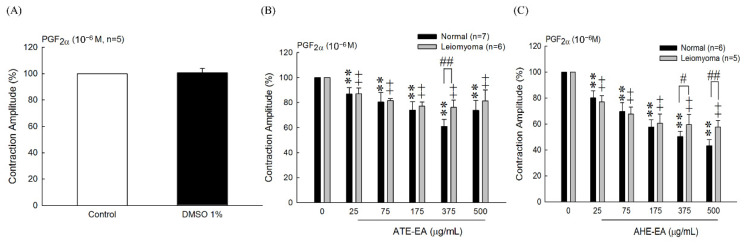
Effects of DMSO (**A**) and serial concentrations of ATE-EA (**B**) and AHE-EA (**C**) on PGF_2α_-induced contraction amplitude on both normal myometrial tissue and leiomyoma tissue, respectively. Data represent mean values ± SD. ** *p* < 0.01 compared with control. # *p* < 0.05, ## *p* < 0.01 compared with normal human uterine tissue. PGF_2α_, prostaglandin F2 alpha; ATE-EA, ethyl acetate fraction of adlay testa ethanolic extract; AHE -EA, ethyl acetate fraction of adlay hull ethanolic extract.

In 2008, Hsia et al. reported that 1% DMSO as solvent had no effect on the PGF_2α_-induced uterine contractions [22]. Our study’s result showed that DMSO 1% had no effect on human uterine contraction (Figure 6A). Moreover, the inhibitory effect of ATE-EA or AHE-EA, at increasing concentrations, was better on normal myometrial tissue than that of leiomyoma (Figure 6B,C). Interestingly, ATE-EA at high doses of 375 µg/mL and AHE-EA (375 and 500 μg/mL) exerted significant inhibitory effects on normal human uterine tissue contraction compared with human leiomyoma tissues both induced by PGF_2α_ as shown in (Figure 6B) and (Figure 6C), respectively. 

### 3.7. Effect of ATE-EA and AHE-EA on Oxytocin-Induced Writhing Test

As shown in Table 2, results from oxytocin-induced writhing in mice showed that ATE-EA and AHE-EA significantly inhibited the OT-induced writhing. The amount of writhing after oral administration of the ATE-EA and AHE-EA were significantly lower than that of the model group within 30 min. Mice treated with ATE-EA, AHE-EA, and ibuprofen showed latency increase induced by the oxytocin test.

### 3.8. Effect of ATE-EA and AHE-EA on Acetic Acid-Induced Writhing Test

The antinociceptive effect of ATE-EA and AHE-EA on the writhing test in mice is shown in Table 3. ATE-EA significantly reduced writhing response compared to the control group, but AHE-EA only slightly decreased the trend.

### 3.9. Analysis of Flavonoids of ATE-EA and AHE-EA by HPLC and HPLC-MS

**Figure 7:**
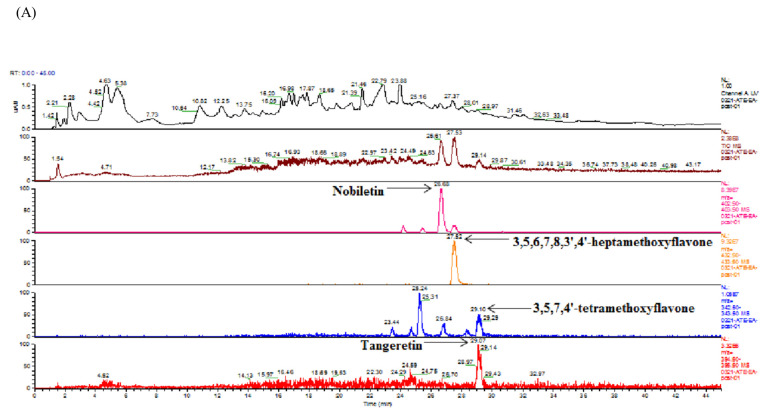

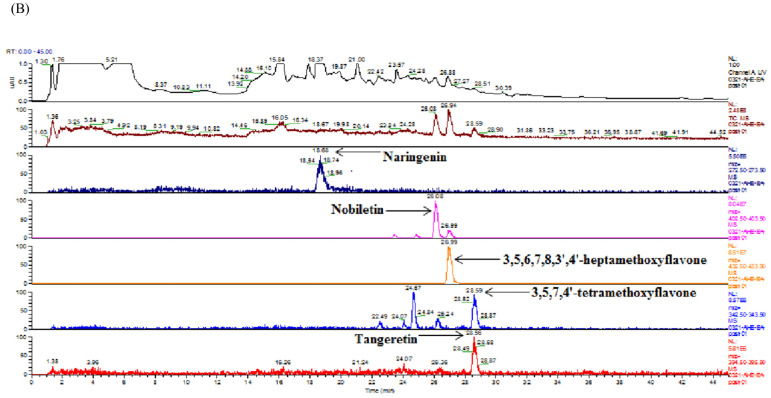
Total ion chromatogram of extracts from adlay (**A**) ATE-EA and (**B**) AHE-EA.

The ATE-EA and AHE-EA were analyzed using HPLC at 280 and 350 nm. Five flavonoid compounds (3,5,6,7,8,3′,4′-heptamethoxyflavone, 3,5,7,4′-tetramethoxyflavone, nobiletin, tangeretin, and naringenin) were identified from ATE-EA and AHE-EA by matching their retention times against those of the standards. Table 4 shows the quantification of five flavonoids of ATE-EA and AHE-EA, while Figure 7 shows the ATE-EA and AHE-EA chromatograms. The spectrum of the standard of flavonoids in positive model with the HPLC-ESI (+)/MS is shown in Figure S1.

### 3.10. Analysis of Fatty Acid and Phytosterol of ATE-EA and AHE-EA by GC

Table 5 shows the results of ATE-EA and AHE-EA analysis of fatty acids by gas chromatography. Both main compounds were four fatty acids, namely, palmitic acid, linoleic acid, stearic acid, and oleic acid, of quantity in order. Phytosterol of ATE-EA and AHE-EA consisting of campesterol, stigmasterol, and β-sitosterol are shown in Table 6.

## 4. Discussion

Most experimental and clinical research has reported that pathogenesis of primary dysmenorrhea identified uterine prostaglandins as a substantially contributing factor within the last 15 years [27]. PGF_2α_ is the principal prostaglandin in the uterus, which functions to increase the contractility of the myometrium. Previous studies have been reported that PGF_2α_ plays an important role in parturition, including increased contractility, amplitude, duration, and frequency of spontaneous contractility as a myometrial stimulant [28]. The cause for dysmenorrhea is tissue ischemia resulting from the vessel’s constriction, an increase of intrauterine pressure, and a decrease of uterine blood flow. Thus, prostaglandin theory is a convincing piece of evidence for achieving pain relief of inhibition prostaglandin synthesis and decreased intrauterine pressure [27]. It has been demonstrated that various mechanisms regulate smooth muscle contractility by changing the intracellular calcium contraction ([Ca^2+^]i) [29,30]. The extracellular Ca^2+^ release and Ca^2+^ influx are mediated by phospholipase C activation to IP3 generation, and the receptor-operated Ca^2+^ channels (ROC) resulted in ATP-induced [Ca^2+^]i increase [31]. Meanwhile, voltage-operated Ca^2+^ channels (VOC) are another factor predominantly responsible for the increase in the [Ca^2+^]i, resulting in membrane depolarization to Ca^2+^ influx such as high K^+^-induced contractions. Oxytocin coupled to the receptor to stimulate inositol trisphosphate (InsP3) production for contraction as well as PGF_2α_ does not directly release Ca^2+^ from store sites [32]. The experiments were designed to investigate the potential inhibition induced by agonists such as PGF_2α_, oxytocin, carbachol, or K^+^ solution produced contractions by adlay ethanolic extracts. We then further examined the effect of four parts and their subfractions of ethanolic extracts of adlay in vitro and in vivo. We previously found that methanolic extracts of adlay hull inhibited uterine contractions induced by PGF_2α_ by blocking external Ca^2+^ influx [22]. Coix seed oil could prevent or reduce the contracture action of striated muscle, as previously described by Liu [33]. Consistent with previous reports [20,22], our results suggested that the ATE-EA and AHE-EA from ethanolic extracts of adlay strongly suppressed smooth muscle by blocking ROCs or VOCs while inhibiting extracellular calcium ions influx.

When examining the effect of ATE-EA and AHE-EA in modulating smooth muscle contraction for protection, we found the presence of ROC and VOC drugs to increase cumulative concentration. The current findings showed PGF_2α_ or oxytocin-induced contraction of the AHE-EA was a better strongly protective effect of smooth muscle hypercontraction than ATE-EA. However, ATE-EA and AHE-EA had no protective effect on the carbachol-mediated contraction. A competitive antagonist blocks the chain of reactions produced by an agonist, as the antagonist acts on a different site in the receptor within the effector system in pharmacology [34]. Thus, the ATE-EA supposed aspect of inhibitory effect might have a less active compound, quercetin, in comparison with AHE-EA in terms of explaining the observed effect. These results are similar to our previous study, which indicated that naringenins are the major pure components of AHM-EA in terms of suppressing contraction [22]. This is reported in patients with uterine leiomyoma complaints, including menorrhagia, dysmenorrhea, pain, and mild renal impairment [35]. Moreover, the effect of 375 μg/mL ATE-EA and AHE-EA showed a consistent and highly significant reduced contractility of human leiomyoma and normal myometrial smooth muscle.

Chrysoeriol (luteolin 3′-methyl ether), a bioactive flavonoid, is found in several tropical, medicinal plants known for potent antioxidant [36], anti-inflammatory, antitumor, antimicrobial, and antiviral activity. In the literature, some reports show selective bronchodilator effect by K^+^-induced contractions in the trachea and aorta [37], as well as smooth muscle-relaxing effects [38]. No data to date are available regarding the question of whether 3,5,6,7,8,3′,4′-heptamethoxyflavone inhibits contractility on uterine smooth muscle. This study is the first report showing that 3,5,6,7,8,3′,4′-heptamethoxyflavone has an inhibitory effect on uterine contraction.

The oxytocin-induced writhing response preserved for about 7 days was used to evaluate clinical features of primary dysmenorrhea in estrogen-treated mice. When oxytocin was given within 0~30 min, 90% of the twisting body reacted with concentration. This model for dysmenorrhea in mice was simple, reliable, and economical [24]. In the present study, we demonstrated that ATE-EA and AHE-EA inhibited the contraction of isolated uterus induced by oxytocin. In addition, we demonstrated that ATE-EA and AHE-EA significantly reduced oxytocin-induced writhing response. Our results were similar to those reported previously [39,40]. Taken together, these data suggest that ATE-EA and AHE-EA may have potential therapeutic agents for dysmenorrhea.

Administration of acetic acid of writhing resulted in profound pain of endogenous nature for a prolonged period of time because writhing induced the overt response by nociceptors. The writhing characterized the retraction of the abdomen and stretching of hind limbs. The writhing test is the animal model commonly used to evaluate the analgesic effect [41]. Collier et al. reported that writhing might partly result from stimulation of prostaglandin biosynthesis [42] as well as the mechanism accounting for the efficacy of non-steroidal anti-inflammatory drugs. Treatment with coix seed oil for cancer patients effectively reduced the efficacy of pain relief in a clinical study [43]. Adlay also was found to exert many functions, such as anticancer effects, blood sugar reduction, and pain relief, for human health [12]. In this study, ATE-EA alone exhibited antinociceptive activity in the acetic acid-induced writhing test. In addition, ATE-EA may have more antinociceptive activity than AHE-EA in mice. Further studies are needed to elucidate the mechanism of the antinociceptive action of ATE-EA and AHE-EA.

## 5. Conclusions

In conclusion, both ATE-EA and AHE-EA provide clear evidence for the relaxant effects on rat uterus and human uterine tissue when administered in an animal model of the oxytocin-induced writhing test. Therefore, these findings suggest that ATE-EA and AHE-EA may be a new strategy for reducing strong uterine contraction for dysmenorrhea. The possible underlying mechanisms of ATE-EA and AHE-EA could be due to the blockage of calcium influx of extracellular calcium in rat uterus.

## Figures and Tables

**Table 1:** Inhibitory activities of flavonoids on PGF2α-induced uterine contractions of rat ex vivo.

Compound	IC_50_ (μM)
Chrysoeriol	25.59
Formononetin	33.11
Biochanin A	37.04
3,5,6,7,8,3′,4′-Heptamethoxyflavone	24.91
Farrerol	37.03
3,5,7,4′-Tetramethoxyflavone	38.91
Isoliquiritigenin	55.34
3,5,7,3′,4′-Pentamethoxyflavone	38.4
Nobiletin	46.43
Homoeriodictyol	85.86
Tangeretin	96.7
Naringenin	147.1
Liquiritigenin	176.8

The concentration that caused 50% inhibition (IC_50_) is given as the mean ± SD of quadruplicate experiments.

**Table 2:** Effect of ATE-EA and AHE-EA on the oxytocin-induced writhing response in estrogen-treated mice. The latent period and number of writhes was counted for 30 min after oxytocin administration.

Group	Latent Period (s)	Writhing Numbers
Control	1800 ± 0 ^a^	0.0 ± 0.0 ^c^
Model control	416 ± 293 ^c^	12.9 ± 4.6 ^a^
ATE-EA	1057 ± 553 ^b^	1.4 ± 1.0 ^bc^
AHE-EA	1459 ± 547 ^ab^	4.2 ± 4.3 ^b^
Ibuprofen	1678 ± 322 ^a^	0.6 ± 1.1 ^c^

The data are presented as mean ± SD (n = 8) and analyzed with one-way ANOVA with Duncan’s multiple comparison test. Different letters indicate significant differences among groups at the level of *p* < 0.05. Mode control, treated with stilbestrol.

**Table 3:** Effect of ATE-EA and AHE-EA on the acetic acid writhing response in acetic acid-treated mice. The latent period and umber of writhes was counted for 30 min after oxytocin administration.

Group	Latent Period (s)	Writhing Numbers
Control	268 ± 74 ^a^	30.8 ± 8.6 ^a^
ATE-EA	402 ± 85 ^b^	16.2 ± 5.4 ^b^
AHE-EA	343 ± 58 ^ab^	34.1 ± 11.8 ^a^
Aspirin	500 ± 84 ^c^	6.7 ± 3.4 ^c^

The data are presented as mean ± SD (n = 8) and analyzed with one-way ANOVA with Duncan’s multiple comparison test. Different letters indicate significant differences among groups at the level of *p* < 0.05.

**Table 4:** Chemical composition analysis of flavonoid compounds of ATE-EA and AHE-EA by HPLC.

Compound (μg/g)	AHE-EA	ATE-EA
Naringenin	8053.74 ± 71.23	N.D.
Tangeretin	1210.51 ± 26.32	352.55 ± 9.03
3,5,7,4′-Tetramethoxyflavone	219.83 ± 5.31	62.9 ± 10.95
Nobiletin	67.16 ± 4.54	719.42 ± 6.98
3,5,6,7,8,3′,4′-Heptamethoxyflavone	N.D.	N.D.

N.D.: non-detection.

**Table 5:** Chemical composition analysis of fatty acid of ATE-EA and AHE-EA by GC.

Compound (mg/g)		AHE-EA	ATE-EA
C8:0	Caprylic acid	N.D.	N.D.
C10:0	Capric acid	N.D.	N.D.
C12:0	Lauric acid	0.38	0.41
C14:0	Myristic acid	0.29	0.62
C14:1	Myristoleic acid	0.29	0.41
C16:0	Palmitic acid	17.38	33.53
C16:1	Palmitoleic acid	N.D.	0.62
C18:0	Stearic acid	2.5	4.55
C18:1	Oleic acid	43.2	95.84
C18:2	Linoleic acid	23.9	48.85
C18:3	γ-Linoleic acid	N.D.	N.D.
C18:3	α-Linoleic acid	0.58	1.45
C20:0	Arachidic acid	0.67	1.66
C20:1	Eicosaenoic acid	0.29	0.62
C20:2	Eicosadienoic acid	N.D.	0.41
C20:3	γ-Eicosatrienoic acid	N.D.	N.D.
C20:3	α-Eicosatrienoic acid	N.D.	1.66
C20:4	Arachidonic acid	N.D.	N.D.
C20:5	Eicosapentaenoic acid	N.D.	N.D.
C22:0	Behenic acid	0.67	1.86
C22:1	Erucic acid	N.D.	N.D.
C22:2	Docosadienoic acid	N.D.	N.D.
C22:4	Docosatetraenoic acid	N.D.	N.D.
C22:5	Docosapentaenoic acid	N.D.	N.D.
C22:6	Docosahexaenoic acid	N.D.	N.D.
C24:0	Lignoceric acid	1.15	4.35
C24:1	Nervonic acid	N.D.	0.21
	Others	4.61	9.73

N.D.: non-detection.

**Table 6:** Chemical composition analysis of phytosterol of ATE-EA and AHE-EA by GC.

Compound (μg/g)	AHE-EA		ATE-EA	
Brassicasterol	N.D.		N.D.	
Campesterol	407.20	±2.64	1017.77	±2.21
Stigmasterol	515.09	±5.28	1872.43	±9.16
β-Sitosterol	2315.68	±5.97	3286.61	±13.80
Stigmastanol	N.D.		3.52	±0.21
Δ^5^-Aveasterol	6.61	±0.21	8.35	±0.23
Δ^7^-Stigmasterol	N.D.		14.03	±0.76

N.D.: non-detection.

## Data Availability

Not applicable.

## Supplementary Materials

The following are available online at https://www.mdpi.com/article/10.3390/biom11060887/s1, Figure S1: The chromatogram of the standard of flavonoids in positive model with the HPLC-ESI (+)/MS. Table S1: The retention time, molecular weight and mass signal of the flavonoid compounds in AHE-EA and ATE-EA.
