# Supplementary material for: Ethanolic Extracts of Adlay Testa and Hull and Their Active Biomolecules Exert Relaxing Effect on Uterine Muscle Contraction through Blocking Extracellular Calcium Influx in Ex Vivo and In Vivo Studies
Source: Biomolecules. 2021 Jun 15;11(6):887. doi: 10.3390/biom11060887 (PMC8232605; doi:10.3390/biom11060887)
Supplement: Supplementary file 1 [file biomolecules-11-00887-s001.zip › biomolecules-1252255-supplementary.pdf]

Ethanolic extracts of adlay testa and hull and their active biomolecules exert relaxing effect on uterine muscle contraction through blocking extracellular calcium influx in ex vivo and in vivo studies

Yun-Ju Huang[1], Yu-Chieh Chen[2], Hsin-Yuan Chen[1], Yi-Fen Chiang[1], Mohamed Ali[3], Wenchang Chiang[2], Cheng-Pei Chung[4], Shih-Min Hsia[1,5,6,7*]

1 School of Nutrition and Health Sciences, College of Nutrition, Taipei Medical University, Taipei 11031, Taiwan; d04641004@ntu.edu.tw (Y.-J.H.); hsin246@gmail.com (H.-Y.C); yvonne840828@gmail.com (Y.-F.C.)

2 Institute of Food Science and Technology, National Taiwan University, Taipei 10617, Taiwan; r00641033@ntu.edu.tw (Y.-C.C); chiang@ntu.edu.tw(W.C.)

3 Clinical Pharmacy Department, Faculty of Pharmacy, Ain Shams University, 11566 Cairo, Egypt; mohamed.aboouf@pharma.asu.edu.eg (M.A.)

4 Department of Nutrition and Health Sciences, Chang Gung University of Science and Technology, Taoyuan 33303, Taiwan.

5 Graduate Institute of Metabolism and Obesity Sciences, College of Nutrition, Taipei Medical University, Taipei 11031, Taiwan.

6 School of Food Safety, College of Nutrition, Taipei Medical University, Taipei 11031, Taiwan.

7 Nutrition Research Center, Taipei Medical University Hospital, Taipei 11031, Taiwan

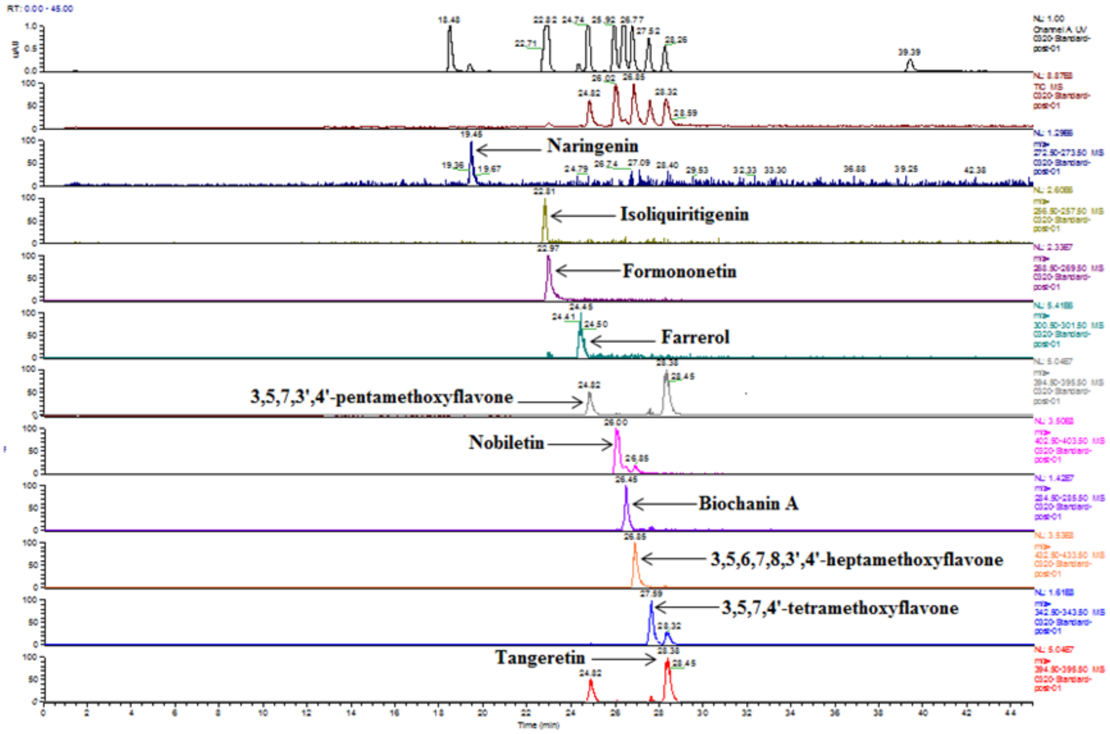

Figure S1. The chromatogram of the standard of flavonoids in positive model with the HPLC-ESI (+)/MS.

Table S1. The retention time, molecular weight and mass signal of the flavonoid compounds in AHE-EA and ATE-EA

Compound	MW	Retention time (min)	Mass signal (m/z)
Chrysoeriol	300	N.D.	
Formononetin	268	22.97	269 $[M+H]^+$
Biochanin A	284	26.45	285 $[M+H]^+$
3,5,6,7,8,3′,4′-heptamethoxyflavone	432	26.85	433 $[M+H]^+$
Farrerol	300	24.45	301 $[M+H]^+$
3,5,7,4′-tetramethoxyflavone	342	27.59	343 $[M+H]^+$
Isoliquiritigenin	256	22.81	257 $[M+H]^+$
3,5,7,3′,4′-pentamethoxyflavone	372	24.82	395 $[M+Na]^+$
Nobiletin	402	26.00	403 $[M+H]^+$
Homoeriodictyol	302	N.D.	
Tangeretin	372	28.38	395 $[M+Na]^+$
Naringenin	272	19.45	273 $[M+H]^+$
Liquiritigenin	256	N.D.	

N.D.: non-detection
